# Cruciate Substituting Implants in Primary Total Knee Arthroplasty

**DOI:** 10.1155/2022/2676715

**Published:** 2022-01-24

**Authors:** Alexander J. Volkmar, Robert Elrod, Justin W. Vickery, Charlie C. Yang, Gregory G. Polkowski, J. Ryan Martin

**Affiliations:** ^1^Department of Orthopaedic Surgery, Vanderbilt University Medical Center, Nashville, TN 37232, USA; ^2^Colorado Joint Replacement, Denver, CO 80210, USA

## Abstract

The use of cruciate substituting (CS) total knee replacement has been increasing in popularity. There are numerous factors that have likely contributed to this expansion. The CS philosophy incorporates the ease of use commonly cited by advocates of the posterior stabilized (PS) total knee design with the bone preservation associated with a cruciate retaining (CR) design. The ultra-congruent highly cross-linked polyethylene liner increases stability without an appreciable change in wear. Furthermore, balancing the flexion and extension gaps does not require “titrating” the posterior cruciate ligament, improving the user-friendliness. This paper reviews the nuances of this implant design compared to PS and CR designs as well as provides surgical technique recommendations/considerations.

## 1. Introduction

The use of cruciate substituting (CS) total knee arthroplasty (TKA) has been around for decades [1, 2]. However, initial results have been mixed, and widespread use was therefore limited. This is likely related to early studies demonstrating limited femoral rollback due to implant design features as well as fears of increased polyethylene wear [3]. Furthermore, there were concerns for increased posterior subluxation of the tibia. This was due to the absence of the posterior cruciate ligament (PCL) and decreased femoral tibial congruency of the implant design [4]. For this reason, many surgeons continued to limit use of CS implants and have preferred cruciate retaining (CR) and/or posterior stabilized (PS) total knee implants instead.

Widespread use of contemporary highly cross-linked polyethylene has largely eliminated concerns of wear-related failure [5]. The improved wear characteristics have led to greater confidence in highly congruent tibial polyethylene inserts. While this has contributed to the increased use of CS implants, a variety of other factors have also contributed. The CS implant represents an ideal combination of the theoretical benefits of the PS and CR designs. The CS implant does not rely on the PCL for balancing the flexion gap, and therefore late rupture or attenuation will not impact long-term implant stability. Additionally, the lack of a femoral box decreases the incidence of crepitus and potentially decreases the risk of a femoral condyle fracture seen more frequently with PS implants [6].

Optimizing operating room efficiency becomes more important as the total number of patients undergoing TKA continues to increase, and techniques that facilitate quick, cost-effective procedures are becoming more important. This includes using polyethylene implants that are independent of PCL functionality and do not require an additional femoral box cut. Another method to maximize cost reduction and turnover efficiency that has been proposed is the use of single use implants [7]. The CS implant should be considered as one of the three options of primary total knee arthroplasty implants. This implant can be used with any methodology of implant placement including gap-balancing, measured resection, kinematic alignment, computer navigation, and/or robotic-arm assisted TKA. Additionally, cemented and cementless fixation can be utilized. The following surgical technique will discuss the nuances of utilizing a primary CS design as well as compare this with both PS and CR techniques. This paper should serve as a reference for surgeons that are considering transitioning from a PS or CR implant design to a CS.

## 2. Main Text

### 2.1. Primary CS Protocol

The skin incision is commonly made in a linear and vertically oriented manner, just medial to the tibial tubercle and extended proximally 2–4 inches proximal to the proximal pole of the patella. Any arthrotomy can be utilized; however, the authors prefer to use a standard medial parapatellar approach. The sequence of ligamentous releases and bony resections is surgeon dependent, and the use of a CS implant should not necessarily change this process. The authors prefer a gap-balancing technique; however, measured resection, kinematic alignment, and technology-assisted techniques can easily be applied.

In most instances, and especially with gap-balancing techniques, early release of the PCL should be considered. Late release of the PCL can increase the flexion gap, which may require subsequently increasing the extension gap to achieve symmetry. However, in measured resection techniques, late release can be considered. Alternatively, in surgeons that are considering transitioning to this implant from a CR methodology, release of the PCL to balance the flexion/extension gap and use of an ultra-congruent liner could be considered rather than transitioning to a PS implant.

### 2.2. Femoral Considerations

The distal femoral cut and method for determining implant rotation of the femoral component are not dependent on the choice of a CS implant. Therefore, surgeons that prefer either CR or PS implants can utilize their preferred method (gap-balancing, measured resection, and so on). However, no box cut is necessary. The lack of a femoral box cut decreases the risk for femoral condyle fracture that can be associated with PS implants [6, 8]. In patients with smaller implant sizes, a large femoral box may put patients at risk for this rare complication. This may be a particular consideration for PS users that are considering transitioning to a CS implant. PS users may also consider a CS construct in patients that have distal femoral hardware in place that would preclude the use of a femoral box.

One additional consideration for PS users that are considering transitioning to a CS design is the femoral component dimensions. Some implant manufacturers decrease the posterior femoral condylar offset in their cruciate retaining/substituting femoral components. This should be factored into the balancing of flexion and extension gaps when transitioning from PS to CS techniques to avoid a potential asymmetry.

### 2.3. Tibial Considerations

The coronal alignment of the tibia is not necessarily dependent on the implant choice of CS, PS, or CR. However, sagittal alignment changes may be necessary in surgeons transitioning from CR to CS. Traditionally, the tibial resection, when utilizing a CR design, would be to cut more posterior slope. This increases the flexion gap and potentially improves knee flexion. However, with PCL resection, excessive slope may lead to flexion instability, depending on when the PCL is released. Therefore, we would recommend early resection of the PCL and a more neutral tibial resection from 0–3°. The choice of implant bearing, rotating platform and fixed bearing, can both be utilized with a CS construct.

### 2.4. Patellar Considerations

While there is a decreased incidence of patellar crepitus with knee designs that do not have an intercondylar box, patellar resurfacing is at the discretion of the surgeon and implant choice should not impact this decision [9].

## 3. Discussion

Our understanding of native knee kinematics has evolved substantially over the last several decades. Initial emphasis was placed on the concept of femoral rollback and posterior translation of the femur on the tibia in the sagittal plane as the knee flexes. More recently, additional attention has been placed on restoration of axial rotation and posterior translation of the lateral femur as the knee enters deep flexion [10, 11]. This motion has been referred to as “medial pivot” [12].

While CR, PS, and CS implants have been utilized for years, the CS implants were initially not favorable due to earlier designs using ultra-congruent implants on standard cross-linked polyethylene. Additionally, one early fluoroscopic study demonstrated reduced femoral rollback and inferior range of motion with this design [3]. Furthermore, the congruity of the implant led to concerns regarding increased polyethylene wear. Therefore, widespread use did not occur.

More recent studies comparing CS implants to PS or CR implants have shown favorable results. Wautier and Thienpont demonstrated anteroposterior laxity at 30° and 90° of flexion for two PS TKA designs, which was not seen in a medial pivot CS implant [13]. However, all three implants showed anteroposterior laxity at 60° of flexion [13]. Lützner et al. also compared PS implants with CS implants in a randomized controlled trial of 127 patients and found that the CS design resulted in increased intraoperative sagittal translation and reduced posterior femoral rollback during knee flexion [14].

In terms of clinical outcomes of these implants, results have been largely positive, showing either no clinical differences or slightly better outcomes with CS liners. The study mentioned previously by Wautier and Thienpont showed no difference in either patient reported outcome measurement scores or proprioception [13]. Lützner et al. found no difference in knee's range of motion, intraoperatively or at follow-up. However, they demonstrated significantly improved Oxford Knee Scores in patients who received CS liners at 1 year follow-up [14]. This corresponds with the positive results shown by Samy et al., who found no difference in range of motion comparing PS or CS liners but found that patients receiving CS liners scored better in Forgotten Joint Score, particularly in deep flexion [12].

The stability offered by the augmented congruity of the femur with the CS insert may be substantially greater than traditional inserts, particularly in anteroposterior translation and tibial internal rotation [15]. Song et al. prospectively evaluated 76 patients treated with either CR or CS liners. There were no differences between groups in mediolateral or anteroposterior laxity measured using stress radiographs [16]. This replicates the findings of a similar study by Lützner et al. which concluded that CS liners offer similar stability compared to CR liners, without the need to resect as much bone from the distal femur [17]. Studies comparing clinical outcomes of CS and CR liners are similar to the clinical outcomes seen comparing CS and PS liners, with no significant differences seen in postoperative range of motion, Hospital for Special Surgery score, Knee Society score, and Western Ontario and McMaster Universities Osteoarthritis Index subscale score [16].

## 4. Implementation into Practice

There has been an increase in interest for CS total knee replacement designs. It appears to be a happy middle ground for surgeons who prefer CR or PS techniques. Surgeons who currently utilize a PS implant might find the lack of an intercondylar box to be appealing. Eliminating this bony resection decreases the risk of femoral condyle fractures and lowers the incidence of patellar crepitus. This potential risk of femoral condyle fractures is most likely to occur with a combination of smaller femoral implant sizes in conjunction with a PS implant that has a large intercondylar box design [8]. Alternatively, surgeons who commonly utilize a CR design might prefer the ability to sacrifice the PCL without jeopardizing implant stability due to the congruency of certain CS designs.

Transitioning from a PS or CR methodology to a CS implant can be a very seamless process and may contribute to shorter operating time, as it removes the necessity to cut a femoral box. In the randomized controlled trial by Lützner et al. mentioned above, they noted a 7-minute shorter operating time with the CS design.

Surgeons transitioning from CR to CS implant will note a similar ease of transition. The ability to balance the knee flexion and extension gaps without relying on a potentially attenuated PCL can increase the reproducibility of CS knees. Furthermore, the implants for CR and CS are typically similar except for the polyethylene insert having increased congruity compared to the CR implant. The authors' two primary recommendations for CR users that are considering transitioning to a CS implant would be to resect the PCL early and to cut less tibial slope. These two recommendations will be helpful in avoiding an excessive flexion gap.

## 5. Conclusion

The CS total knee implant should be considered as one of the three primary total knee arthroplasty implant choices. With modern implant design features including ultra-congruent highly cross-linked polyethylene, the contemporary CS implant has statistically similar outcomes to PS implants. However, the CS has a lower incidence of patellar clunk and is bone preserving. The CS implant offers an ideal mix of the benefits of both CR and PS designs and can be easily incorporated into any surgical workflow.

## Data Availability

The data supporting this review are from previously reported studies and datasets, which have been cited within the article.
